# Serum Homocysteine Levels in Men with and without Erectile Dysfunction: A Systematic Review and Meta-Analysis

**DOI:** 10.1155/2018/7424792

**Published:** 2018-08-07

**Authors:** Andrea Sansone, Angelo Cignarelli, Massimiliano Sansone, Francesco Romanelli, Giovanni Corona, Daniele Gianfrilli, Andrea Isidori, Francesco Giorgino, Andrea Lenzi

**Affiliations:** ^1^Department of Experimental Medicine, Section of Medical Pathophysiology, Food Science and Endocrinology, Sapienza University of Rome, Rome, Italy; ^2^Department of Emergency and Organ Transplantation, Section of Internal Medicine, Endocrinology, Andrology, and Metabolic Diseases, University of Bari Aldo Moro, Bari, Italy; ^3^Endocrinology Unit, Medical Department, Azienda Usl Bologna Maggiore-Bellaria Hospital, Bologna, Italy

## Abstract

**Objectives:**

Elevated levels of serum homocysteine (Hcy) have been associated with cardiovascular diseases and endothelial dysfunction, conditions closely associated with erectile dysfunction (ED). This meta-analysis was aimed to assess serum Hcy levels in subjects with ED compared to controls in order to clarify the role of Hcy in the pathogenesis of ED.

**Methods:**

Medline, Embase, and the Cochrane Library were searched for publications investigating the possible association between ED and Hcy. Results were restricted by language, but no time restriction was applied. Standardized mean difference (SMD) was obtained by random effect models.

**Results:**

A total of 9 studies were included in the analysis with a total of 1320 subjects (489 subjects with ED; 831 subjects without ED). Pooled estimate was in favor of increased Hcy in subjects with ED with a SMD of 1.00, 95% CI 0.65–1.35, *p* < 0.0001. Subgroup analysis based on prevalence of diabetes showed significantly higher SMD in subjects without diabetes (1.34 (95% CI 1.08–1.60)) compared to subjects with diabetes (0.68 (95% CI 0.39–0.97), *p* < 0.0025 versus subgroup w/o diabetes).

**Conclusions:**

Results from our meta-analysis suggest that increased levels of serum Hcy are more often observed in subjects with ED; however, increase in Hcy is less evident in diabetic compared to nondiabetic subjects. This study is registered with Prospero registration number CRD42018087558.

## 1. Introduction

Prevalence of erectile disorders increases steadily with aging, from 1 to 10% in men younger than 40 years up to 70–100% in men older than 70 years [1, 2]. It is widely accepted that several factors might be involved in the pathogenesis of erectile dysfunction (ED): guidelines suggest investigating organic causes [3], such as endocrine alterations [4, 5], neurological impairment [1, 6], and vascular dysfunction [6] as well as psychological or relational issues [7, 8].

However, given the importance of hemodynamics in allowing adequate erection, it should come as no surprise that ED shares the same risk factors of many cardiovascular (CV) affections [9].

In these regards, hyperhomocysteinemia (HHcy) has sprung into attention for its involvement in endothelial dysfunction [10]. Several meta-analysis studies have confirmed the association with consistently elevated levels of homocysteine (Hcy) and the risk of CV diseases: Boushey and colleagues [11] reported a 1.6 OR (95% CI 1.4–1.7) for coronary artery disease in men following an increase of 5 *μ*mol/l in serum Hcy levels, whereas Clarke et al. [12] described that a reduction of 25% of serum Hcy was associated with an 11% lower risk of ischemic heart disease and with a ~20% lower risk of stroke. However, other reports have suggested that Hcy lowering interventions do not prevent CV events [13], or that Hcy is a marker of unhealthy lifestyles [14], thus being indirectly associated with CV health.

Despite the prevalence of ED and its known association with endothelial dysfunction [15, 16], its link with serum Hcy has not been adequately addressed. HHcy is associated with increased arterial stiffness [17] and with impaired nitric oxide synthase activity [18]. Several studies have assessed whether subjects suffering from ED have increased serum Hcy compared to controls, but most of these studies have been performed in small populations and generally do not provide solid evidence in these regards.

## 2. Materials and Methods

### 2.1. Methods

This meta-analysis was performed in line with the Preferred Reporting Items for Systematic Reviews and Meta-Analyses (PRISMA) reporting guideline [19] (see Supplementary file 1). The protocol of this study (CRD42018087558) was published on the website of the University of York (Centre for Reviews and Dissemination) and can be accessed at the following address: http://www.crd.york.ac.uk/PROSPERO/display_record.php?ID=CRD42018087558.

### 2.2. Search Strategy

An extensive Medline, Embase, and Cochrane search was performed, including the following words: (“erectile dysfunction”[MeSH Terms] OR (“erectile”[All Fields] AND “dysfunction”[All Fields]) OR “erectile dysfunction”[All Fields]) OR ((“penile erection”[MeSH Terms] OR (“penile”[All Fields] AND “erection”[All Fields]) OR “penile erection”[All Fields] OR “erectile”[All Fields]) AND (“physiology”[Subheading] OR “physiology”[All Fields] OR “function”[All Fields] OR “physiology”[MeSH Terms] OR “function”[All Fields])) OR ((“sexual behavior”[MeSH Terms] OR (“sexual”[All Fields] AND “behavior”[All Fields]) OR “sexual behavior”[All Fields] OR “sexual”[All Fields]) AND (“physiopathology”[Subheading] OR “physiopathology”[All Fields] OR “dysfunction”[All Fields])) AND (“homocysteine”[MeSH Terms] OR “homocysteine”[All Fields]). The search, which accrued data up to February 28th 2018, was restricted to articles written in English, Italian, or French and studies including human participants. The identification of relevant studies was performed independently by two of the authors (*SA and SM*), and conflicts were resolved by a different investigator (*CA*). We did not employ search software but hand-searched bibliographies of retrieved papers for additional references. The main source of information was derived from published articles.

### 2.3. Study Selection

**Figure 1:**
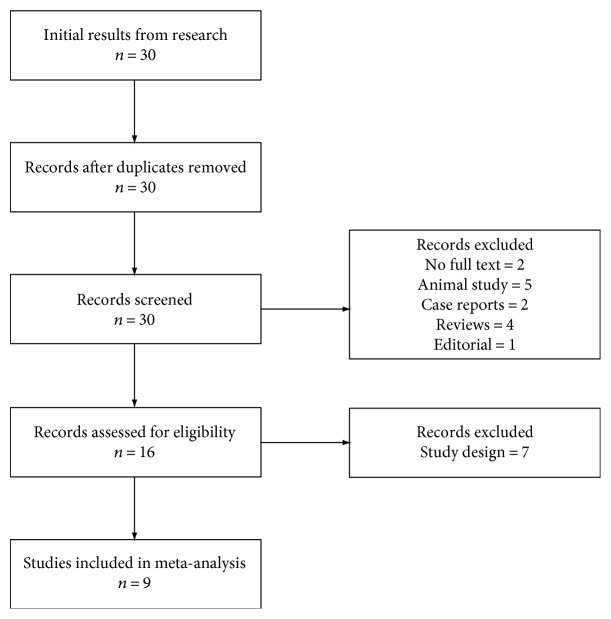
Flowchart detailing all phases of data retrieval.

All studies reporting serum Hcy levels in patients with ED and control population were included. Case reports were excluded from the analysis (see Figure 1).

### 2.4. Outcome and Quality Assessment

The principal outcome was the serum concentration of Hcy in subjects with and without erectile dysfunction. The quality of studies included was assessed using the Cochrane criteria [20]. Difference in Hcy levels between subjects with and without ED was measured with standardized mean difference (SMD) as different kits and tools have been used in different studies (Table 1). All measures of ED were included in the analysis, including the International Index of Erectile Function (IIEF), a 15-item questionnaire extensively used for clinical and research purposes [21], and its abridged, 5-item version (IIEF-5) [22], as well as assessment of ED by means of a single question.

### 2.5. Statistical Analysis

Data from retrieved study were extracted and entered twice by two authors (*SA and CA*) into two separate standard Excel templates which were then cross-checked by the other author. Extracted data included data sources, eligibility, methods, participant characteristics, and results. Heterogeneity in serum Hcy levels was assessed using *I* ^2^ statistics. Even when low heterogeneity was detected, a random effect model was applied, because the validity of tests of heterogeneity can be limited with a small number of component studies. We used funnel plots and the Begg adjusted rank correlation test to estimate possible publication or disclosure bias (Begg et al., 1994); however, undetected bias may still be present because these tests have low statistical power when the number of trials is small. Statistical analysis was performed independently by two authors (*SA and CA*) with the *meta* package on the statistical software R (version 3.4.2) [23].

## 3. Results

Out of 30 retrieved articles, 9 were included in the study (Table 1). The study flow is summarized in Figure 1. The characteristics of the retrieved studies, including assessment of their overall quality, are reported in Table 1 and Supplementary file 1. Retrieved trials included 1320 subjects with a mean age of 39.52 ± 2.11 years. Most of the studies did not provide information in regard to ED severity (missing in 7 studies), BMI of the subjects (missing in 3 studies), smoking habits (missing in 3 studies), and prevalence of diabetes (missing in 1 study).

**Figure 2:**
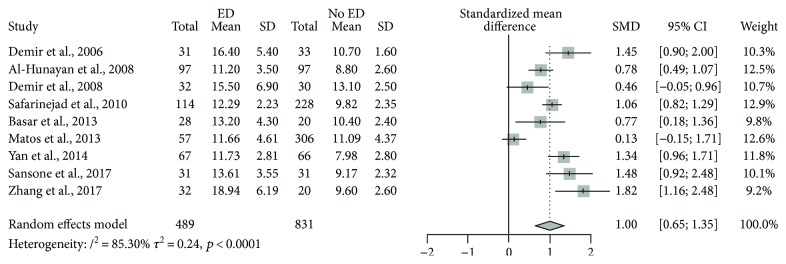
Forest plot for the base model.

**Figure 3:**
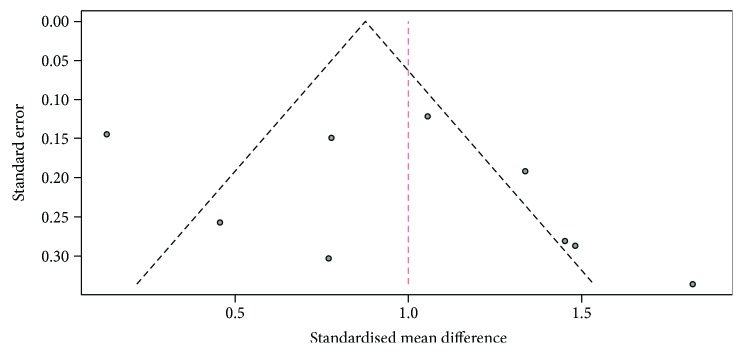
Funnel plot for all studies included in the meta-analysis.

The *I* ^2^ in studies assessing serum Hcy in men with or without ED was 85.3% (*p* < 0.0001, (95% CI 73.9%–91.7%)). Serum Hcy was significantly higher among subjects with ED compared to controls (SMD 1.00, *p* < 0.0001, (95% CI 0.65–1.35)) (Figure 2). A funnel plot and Begg adjusted rank correlation test (Kendall's *τ*: 1.147; *p* = 0.29) suggested no publication bias (Figure 3).

**Figure 4:**
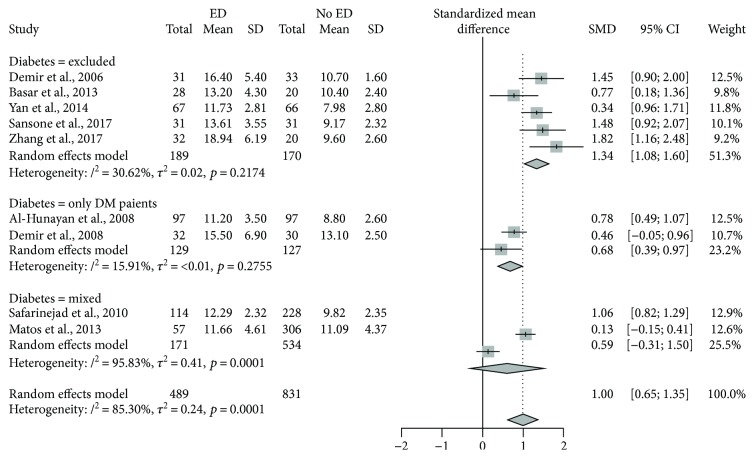
Forest plot by subgroup.

Sensitivity analysis was then performed based on prevalence of diabetes. Five studies [24–28] had diabetes as an exclusion factor, whereas 2 included only subjects with diabetes [29, 30], one [31] did not report actual prevalence of diabetes, and another one [32] reported 4.7% of diabetes among controls and 3.5% among ED patients. The resulting forest plot is shown in Figure 4. Association between Hcy and ED appears steeper in nondiabetic subjects. Indeed, absence of ED is associated with a SMD of 1.34 (95% CI 1.08–1.60) in subjects without diabetes, while SMD is significantly lower in subjects with diabetes (0.68 (95% CI 0.39–0.97); Q 12.00, *p* < 0.0025 versus subgroup w/o diabetes). Despite remaining higher in the subgroup with “mixed” prevalence of diabetes, heterogeneity is remarkably reduced in both other subgroups.

## 4. Discussion

The results of our meta-analysis suggest that Hcy is significantly associated with ED, showing a mean increase of 1.00 standard deviation between controls and ED patients. Among patients with ED, sensitivity analysis has shown that when stratified for presence of diabetes, subjects without diabetes are more likely to have increased levels of serum Hcy compared to diabetic subjects; conversely, this association appears evident in patients with diabetes even for small variation of Hcy, or rather, it could be argued that in diabetic patients, a small increase of Hcy is enough to negatively affect erectile function. In fact, the difference between diabetic and nondiabetic patients might suggest that in the presence of comorbidities, small changes in serum Hcy might negatively be involved in erectile function, based on the premise that multiple risk factors might act in a synergistic, rather than additive, fashion [33, 34]. This observation seems to be rather counterintuitive, as Hcy is a risk factor for cardiovascular events, whose prevalence is higher in diabetic people, and also a sign of metabolic derangement, featured in diabetes [35]. Indeed, different studies observed higher serum level of Hcy both in type 1 and in type 2 diabetes mellitus [36]. More in detail, serum Hcy level appears to be higher in patients affected by T1DM suffering with retinopathy and/or nephropathy [36]. Interestingly, antidiabetic treatment may be considered as an important factor involved in regulation of level of Hcy in diabetic patients [37]: a recent meta-analysis showed both detrimental and beneficial effects of several drugs commonly used in metabolic syndrome and in type 2 diabetes mellitus [38]. Although there seems to be no significant effect of antidiabetic treatments on Hcy levels as a whole (SMD −0.53 (95% CI −1.60 to 0.53)), combination treatment with thiazolidinediones and diguanides or meglitinide and thiazolidinediones has shown significant effects on lowering serum Hcy (SMD −1.67 (95% CI −2.85 to −0.50) and SMD −4.40 (95% CI −4.94 to −3.86), resp.). Following on, metformin appears to increase serum Hcy levels decreasing serum B12 and folate level, although the exact mechanism is not known. Unexpectedly, the improved sensitivity to insulin associated with metformin has been associated to an increase in plasma Hcy [39, 40]; conversely, rosiglitazone as well as pioglitazone showed interesting properties in lowering serum Hcys level [37, 41, 42]. It has been suggested that insulin may stop homocysteine catabolic transformations resulting in an increase in the amount of homocysteine and its blood level [43]. Nevertheless, it should be considered that in T2DM insulin-induced increments of methionine transmethylation, homocysteine transsulfuration, and clearance were markedly reduced [44]. On the contrary, in T1DM, insulin deprivation brings about an increase in Hcy, whereas insulin treatment normalized transsulfuration and remethylation of Hcy, therefore decreasing its serum levels. Furthermore, in the same study, plasma homocysteine concentrations were observed to be lower in T1DM compared to healthy controls [45]. Similarly, in healthy humans, insulin seems to increase in vivo homocysteine clearance [46].

As previously stated, HHcy is closely associated with endothelial dysfunction. Studies in animal models have proven that HHcy markedly inhibits NO formation in rabbit isolated corpus cavernosum [47]. NO acts as a key regulator for endothelial function: activation of guanylyl cyclase by NO leads to increased levels of cyclic GMP (cGMP), which in turn allow for smooth muscle relaxation in the corpora cavernosa [48]. High levels of serum Hcy have been associated with uncoupling of the endothelial NO synthase enzyme (eNOS), therefore reducing availability of NO while at the same time increasing production of reactive oxygen species (ROS). HHcy has also been proven an independent risk factor for atherosclerosis [49], providing further proof of a causative role for Hcy in the pathogenesis of ED.

Our study has some limitations, most notably the small number of studies involved and the lack of a clear definition of ED. A single study [31] assessed presence of ED by means of a single question (“How would you describe your ability to get and keep an erection that is adequate for satisfactory intercourse?”). The remaining studies used validated questionnaires: in detail, four studies [24, 25, 30, 32] used the IIEF and four studies [26–29] used the IIEF-5 [22]. However, most studies did not report separate measurements of serum Hcy based on the degree of severity of ED.

## 5. Conclusions

Results from our meta-analysis suggest that increased levels of serum Hcy are more often observed in subjects with ED: based on existing literature on this topic, a causative role for HHCy as an independent risk factor for ED can be postulated, although confirmation would require interventional studies aimed to decrease serum Hcy levels considering erectile function as primary outcome. Actually, only in rat model of HHcy has been observed an improvement in erectile function after being treated with a demethylation agent [50]. We also reported significantly higher levels of Hcy in subjects without diabetes, compared to diabetic men: while we can assume that this is further proof of a multifactorial pathogenesis for ED, it is also a clear indication that future research in this field should investigate the possible association with other known risk factors—such as smoking habit and obesity—in order to adequately address the possible effects of different variates.

## Figures and Tables

**Table 1:** Studies included in the meta-analysis. Data expressed as means ± standard deviation.

Study	Country	Method	Subjects without ED			Subjects with ED		
			N	Hcy	Age	N	Hcy	Age
Demir et al. [24]	Turkey	Immunoassay	33	10.7 ± 1.6	44.5 ± 4.7	31	16.4 ± 5.4	55.6 ± 8.4
Al-Hunayan et al. [29]	Kuwait	HPLC	97	8.8 ± 2.6	47.6 ± 10.1	97	11.2 ± 3.5	46.6 ± 9.9
Demir et al. [30]	Turkey	Immunoassay	30	13.1 ± 2.5	53.6 ± 6.5	32	15.5 ± 6.9	54.2 ± 7.3
Safarinejad et al. [32]	Iran	HPLC	228	9.82 ± 2.35	31.7 ± 6.6	114	12.29 ± 2.32	32.2 ± 6.4
Basar et al. [25]	Turkey	HPLC	20	10.4 ± 2.4	38.6 ± 8.2	28	13.2 ± 4.3	40.2 ± 7.5
Matos et al. [31]	Brazil	HPLC	306	11.09 ± 4.37	38.6 ± 13.99	57	11.66 ± 4.61	54.1 ± 12.83
Yan et al. [26]	China	Immunoassay	66	7.98 ± 2.8	29.41 ± 4.42	67	11.73 ± 2.81	29.12 ± 4.41
Sansone et al. [27]	Italy	HPLC	31	9.17 ± 2.32	49.14 ± 13.63	31	13.61 ± 3.55	52.83 ± 11.89
Zhang et al. [28]	China	Immunoassay	20	9.6 ± 2.6	32.8 ± 7.8	32	18.94 ± 6.19	34.08 ± 11.99

## Supplementary Materials

Supplementary Materials Characteristics and outcomes of the studies included in the meta-analysis. Y = yes; NA = not applicable; N = no.
